# A Systematic Review of Blockchain for Consent Management

**DOI:** 10.3390/healthcare9020137

**Published:** 2021-02-01

**Authors:** Prasanth Varma Kakarlapudi, Qusay H. Mahmoud

**Affiliations:** Department of Electrical, Computer and Software Engineering, Ontario Tech University, Oshawa, ON L1G 0C5, Canada; qusay.mahmoud@ontariotechu.ca

**Keywords:** blockchain, consent management, privacy

## Abstract

Blockchain technology was introduced through Bitcoin in a 2008 whitepaper by the mysterious Satoshi Nakamoto. Since its inception, it has gathered great attention because of its unique properties—immutability and decentralized authority. This technology is now being implemented in various fields such as healthcare, IoT, data management, etc., apart from cryptocurrencies. As it is a newly emerging technology, researchers and organizations face many challenges in integrating this technology into other fields. Consent management is one of the essential processes in an organization because of the ever-evolving privacy laws, which are introduced to provide more control to users over their data. This paper is a systematic review of Blockchain’s application in the field of consent and privacy data management. The review discusses the adaptation of Blockchain in healthcare, IoT, identity management, and data storage. This analysis is formed on the principles of the Preferred Reporting Items for Systematic Reviews and Meta-Analysis (PRISMA) and a process of systematic mapping review. We provide analysis of the development, challenges, and limitations of blockchain technology for consent management.

## 1. Introduction

Personal data has been added to the long list of precious commodities. Personal information could be anything—a name, an identification number, medical data, or location data. With the world transforming swiftly in terms of technology, every digital activity is registered and collected, which may contain sensitive individual information. According to a survey, there will be approximately 75 billion smart devices by 2025 [1], which will collect user data to monitor. Users give access to these devices to manage their data deliberately or without consent, as they seem reliable, and user profiles are created based on the data provided. This information can be exploited by systems that examine the characteristics and preferences of the user; they attempt to influence decisions by showing content based on aggregated profiles. In 2016, Cambridge Analytica had access to 87 million Facebook users [2], which were acquired via users who were using a third-party application known as “This Is Your Digital Life”, where they unknowingly gave access to the app, which collected their information and their friend’s network information as well. Using extensive data, the company tried to influence then ongoing voters (US Presidential Election 2016) by various means. There are also other incidents where personal data is misused for illicit reasons. So more than anything, it is crucial to understand the policies and the need for data consent, which brings us to an important topic—consent management.

Consent management is the action or process to manage user and customer consent for processing personally identifiable data. In other words, it ensures that users can select and revoke their consent if desired in different categories (preferences). The main steps in this process are collecting consent from users and storing the consent in a secure database, which allows users to withdraw or revoke and renew their consent. While the process seems simple, it has many challenges, especially for big organizations as individual data is scattered around their business, sitting in company silos. Consent is usually collected using a variety of techniques (websites, mobile applications, and various marketing platforms), making it very difficult to aggregate data (map the data collected to a single person). Once aggregated, the next challenge is to store the data in a single repository (a secure database). Data transparency is another challenge that needs to be focused on, as users do not have the perfect knowledge about how their data is being operated. Organizations should also ensure that proper resources are available so that users can see and modify their data preferences. Identity management is a factor that needs to be considered while collecting the user’s consent to the integrity of the information provided. The complexity of implementation becomes harder as privacy rules evolve. These are just a few of the technical challenges with consent management. The other challenge is to inform the user of the purpose of obtaining consent and to make the user aware of it. Additionally, there may be users that approve of the processing of personal information for all reasons and activities, in most instances. However, individuals would like to limit their consent, as a result, with consent management, all these requirements must be addressed while ensuring compliance with the privacy laws.

We believe that consent management with Blockchain makes the process easier, more accessible, and more secure; as it is a decentralized technology, it can make the process more transparent and approachable for users. A blockchain is a digital transaction archive that is duplicated and circulated on the blockchain over the entire computer network. It is indeed important to mention that the tech giants and banking sectors have invested billions of dollars in Blockchain [3]. Bitcoin was the first cryptocurrency to use this technology, and later, more cryptocurrencies such as Monero and Dash started using this technology. In 2013, Vitalik Buterin proposed the idea of Ethereum and started operating on 30 July 2015; Hyperledger was launched by Linux Foundation in December 2015 and had received contributions from IBM, SAP Ariba, and Intel [4]. While Bitcoin, Monero, and Dash are referred to as the first generation of Blockchain, Ethereum comes under the second generation of Blockchain; the third generation of Blockchain are applications that have been developed for various sectors such as healthcare, IoT, supply chain management (SCM), identity management, etc. The third section explains how Blockchain helps with the consent management process.

The purpose of this paper is to systematically collect and classify all related Blockchain research papers from a renowned organization (IEEE), and to achieve the desired purpose, we carried out a systematic review of the Blockchain literature to show some useful insights. There are a few insightful analyses of this subject in the literature, our methodologies and objectives vary from one another, and most of the reviews focus on one application of Blockchain (healthcare, education, or data management). We focused on various applications of Blockchain (in the field of consent management) such as healthcare, IoT, identity management, and data storage. In the review conducted by Ali Alammary et al. [5], the prime focus was on the applications of Blockchain in the field of education. Healthcare was the main application of the systematic review that was conducted by Cornelius C. Agbo et al. [6] and Tsung-Ting Kuo et al. [7]. While Fran Casino et al. [8] discussed the various applications of Blockchain, their review was not in the area of consent management. Therefore, to the best of our knowledge, this is the first systematic review covering a few areas in the field of consent management. Our systematic review follows the guidelines set by PRISMA [9]. It is a well-structured research protocol for systematic review ensuring a thorough and impartial analysis of all published peer-reviewed papers that apply to the topic.

The rest of the paper is structured as follows. Consent management and its importance and challenges are presented in Section 2. An overview of blockchain is presented in Section 3. Section 4 presents the research methodology we have followed. The results are presented in Section 5, and a discussion of the challenges and possible solutions are presented in Section 6. Finally, Section 7 concludes the paper and offers ideas for future work.

## 2. Consent Management

Consent is strictly related to a processing purpose, which is the purpose that the personal data of an individual is processed for, such as marketing, analysis, or healthcare [10]. Consent management is a method, procedure, or collection of policies to enable users to specify what information they are willing to allow access to various care providers [11]. The main steps in consent management involve:

1. Collection of consent;
2. Storage of consent;
3. Use of collected consent and data.

### 2.1. Collection of Consent

Consent can be collected from a variety of sources—websites, mobile applications, CRM (Customer Relationship Management) systems, marketing platforms, contact centers, and sales points. The most traditional way of obtaining consent is through terms of conditions/services or CMP (consent management platform). The main challenge is the process of collecting informed consent from the user. For an instance, a patient might be willing to share his data with a physician but not willing to share his data with a medical researcher. In this scenario, the user provided partial consent to share his data. Therefore, the consent management system should also be designed to capture partial consent from the user. Another example could be a user using an IoT device. Initially, the user provided consent for sharing his preferences of a smart device with the service provider but could decide against sharing at any point in time. Considering the change, the system should also allow the user to revoke the consent at any period.

With the introduction of GDPR (General Data Protection Regulation) and other privacy laws in various countries, companies must make sure the user is entirely aware of the consent they are giving. The company must provide precise information regarding the situations where their data will be utilized; in other words, there should be transparency.

### 2.2. Storage of Consent

As indicated above, consent is collected using a variety of methods, the main task after collecting consent is to aggregate the data. Once properly collected, consent should be stored in a single source of truth, which should be a secure database. Data storage is very important, as it should be stored in a secure location. Data should also be modeled based on the requirement.

The period of data storage is another important case, as the data cannot be stored for longer periods, only for the shortest possible time the data must be stored. The organizations usually come up with the period of data storage, and they must also ensure correct and up-to-date information is maintained. According to GDPR, the time for which personal data are kept should be strictly limited to Recital 39 of the GDPR [12], and the data controller shall set limits for deleting information.

### 2.3. Use of Collected Consent and Data

The following questions arise and need to be addressed after receiving and storing the consent:

- How is the obtained consent used for data access regulation?
- Are all policies such as GDPR and CCPA (California Consumer Privacy Act) met?
- Are there sufficient resources to maintain the transparency of data?

Data of a user can be shared with any third-party organizations based on the consent that is collected. Overall, the main challenging part of the consent management system is to get and maintain consent in a customizable, effective, and transparent way. Ensuring all the conditions set out in the various privacy laws is also very important, as failure could lead to severe penalties. Organizations should also inform users of the purpose of their data, and users should also be informed if their data is sold to third parties for marketing/research purposes. By following all these, the process will be very transparent, which, in turn, will help the organization to build trust with its customers. After ensuring all the policies are met, there should also be resources to help the customer if they want to revoke/withdraw their consent.

Failure in following the regulations can lead up to 10 million Euros or two percent of the annual turnover of the company [13]. In the following section, we explained how Blockchain works and how it helps to achieve consent management.

### 2.4. Efficiency Challenges of Current Consent Management

There are multiple data sources such as patient health information, social media data, data collected from the IoT devices, etc. Since all of these data sources contain sensitive information of individual users, they need to be maintained securely. More importantly, we should also have a systematic and transparent consent collection by the present systems. Some challenges identified in the current systems include:

- Methodologies used for consent collection. The current systems are not capable of collecting or requesting specific consent from the users. They use sophisticated single terms and conditions (T&C). Instead of that, consent should be collected for specific purposes, and it must be presented in a simplified manner.
- Data Regulation. After the consent is collected, the data sharing must be regulated to avoid unwanted sharing of the data. Additionally, current systems do not have a standard platform where the data could be shared among them. Another challenge in the present system is the ability to inform the patients when their data is shared. When there is an exchange of information between hospitals or organizations, the users are generally unaware of the data exchange
- Data handling. The existing healthcare infrastructure should be developed to process the huge volumes of data and should make sure accurate information is being collected from the patients. Storing and collecting inaccurate healthcare information could lead to a fatality when the patient is treated.

To avoid challenges, Blockchain can be used to maintain a flexible and transparent consent management system. Its immutability and decentralized features will make the system trustworthy for the users.

## 3. Overview of Blockchain

Before blockchain technology, transactions were recorded using a centralized model that consists of server-side and client-side applications. In 1990, Haber, S and Stornetta, W. S. came up with the idea of a secured chain of time-stamps [14]. They aspired to create a system where the timestamps of a document cannot be tampered with. However, Blockchain’s concept earned its acclaim in 2008 when it was used as a distributed ledger technology in Bitcoin (Bitcoin white paper published in October 2008 [15]). The mysterious Satoshi Nakamoto introduced Bitcoin, where we can trade electronic coins without a centralized party (banks, for example). In a typical transaction, a third party must be involved in transferring money; third parties are not always reliable, as there is a chance of them getting compromised, and there are transfer limits as well; also, additional amounts are charged when a third party is involved. With the introduction of Bitcoin, we can avoid these confinements. Bitcoin is a virtual currency exchange that takes place without a third party between peers. It is immune to counterfeiting and is preserved by complex algorithms.

Around 2014, cryptocurrency exchanges were not the only application of fascinating blockchain technology. It was explored and used in many other fields. Blockchain uses the SHA-256 algorithm to maintain the data changes in the network and asymmetric cryptography to achieve enhanced security.

### 3.1. Working of Blockchain

As mentioned earlier, Blockchain is used to make sure the documents have not been tampered with, it stores the information in terms of blocks. Usually, each block comprises three things:

- Data
- Hash of block
- Hash of the previous block

**Figure 1:**
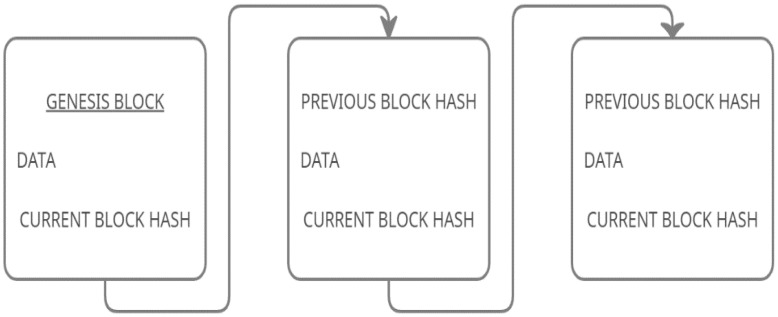
A basic block contains a hash, a previous block hash, and data.

Information stored within a block depends on the Blockchain type; for example, a bitcoin includes financial data (details of a transaction) stored in its blocks. The hash value of a block will be generated when a block is created; its value will not be the same if the data of the block is changed and it also contains the previous block’s hash value. As seen in Figure 1 below, the first block in a blockchain is called a Genesis Block, and it won’t have the hash value of any previous block; instead, it starts from zero.

When there is a new transaction, a block will be added with the corresponding data. Therefore, a new block will be attached, and it establishes a chain in the process, hence the name Blockchain. Due to its immutable property, the data in the Blockchain cannot be modified.

In an instance where data is changed in a block, as the information is changed, its hash value also changes. Therefore, this block’s hash value and the previous hash value of the next blocks will not be the same, resulting in breaking the chain and making it invalid, but there is a chance of recalculating the remaining block’s hash values to make it valid again with the help of advanced supercomputers; to avoid this, we use the concept of proof of work.

The initial consensus algorithm in a Blockchain network is the proof of work (PoW) [16]. PoW is done by people who are called miners to ensure that the transactions made are authentic. This validation method of the transactions is called mining; once transactions are verified valid, they can be added to the Blockchain.

A smart contract is a program that can act as a protocol or an agreement, which cannot be tampered with. This concept is introduced by Nick Szabo in 1994 [17]. In the case of Blockchain, these play a very crucial role. Once deployed on the network, these can be invoked or triggered with the help of a unique address that is assigned to them. Therefore, when a smart contract is deployed, the information is stored on the network, which is accessible to the nodes that are participating. The other nodes on the network cannot change the information on the smart contract. There are many use cases of the smart contract. For instance, when we want to sell a particular object (car, house, etc.,), a potential buyer can be found on the network, thus eliminating the need for a third party. Therefore, smart contracts are self-verifiable. Solidity is one of the main programming languages that is used to create and execute. Ethereum and Zeppelin are Blockchain platforms that use the Solidity programming language.

There are three crucial Blockchain types—public, private, and consortium. All the transactions are made available to the public in public Blockchain; anyone can participate, create, or mine. Private Blockchain or permissioned Blockchain—in this kind, users should have consent to join the group, and it is more centralized than that of public Blockchain. The third kind is consortium blockchain, and it is indistinguishable from private Blockchain, but the key difference between a private and a consortium Blockchain is in a consortium blockchain, it is controlled or regulated by a group instead of a single person, as is the case in a private Blockchain.

### 3.2. Applications of Blockchain Technology

Healthcare. An important issue in the healthcare industry is the privacy of the patients. Patient consent is the key point in the healthcare ecosystem. The technology brings a more secure model for patient information exchange. Considering its distributed ledger feature, it provides more ease of operations between the hospitals. It has the capability to achieve nationwide interoperability between the hospitals for EHR (electronic health records). MedChain [18] was introduced as a data-sharing system. In this system, the data is collected from the medical devices and sensors. There are two events that are created on the network in this model. The first event is created when the healthcare provider adds the data to patient inventory, and the second event is created when a patient provides access to the requestor. Another blockchain application in the medical field is [19], the proposed design is a private blockchain network with Ethereum as its platform and a back end distributed file system. In this prototype, the smart contracts are created as a representation of health records and contain metadata of the ownership, permissions, and data integrity. The prototype deals with various use cases of smart contracts such as the process of issuing the medicines, sharing the lab results with doctors, and for clinical trials, etc.

Internet of Things (IoT). This is will enable businesses to make insights from data collected from IoT devices, which will help them provide improved service to the users. Devices such as Fitbit collect information from the user. In the framework [20], advanced cryptography methods are used to make the design secure and suitable for IoT devices. The prototype deals with off-chain storage, and data is stored on the cloud. The main idea is to have the cloud servers send the hash of the data to the overlay network. When the data is shared to the cloud, the sender adds a digital signature. The overlay network sends an alert to healthcare providers. When the healthcare provider has received an alert, they will have full access to the patient’s data.

Machine Learning. An insurance company could benefit from the integration of ML and blockchain, as identity fraud can be detected before entering the details on the network. In [21], the authors have introduced an algorithm to find the better configurations of weights in a neural network using Blockchain and its concepts. The model was developed on a local network, where the multiple devices are connected. Each node on the network will have the same controls. Transactions can be viewed by each node itself. This avoids unnecessary access to the other nodes. Here, each demand is considered as a transaction, and in the event of a conditional stopping, miners will be awarded based on the better results.

### 3.3. Blockchain for Consent Management

The following comprises an effective consent management system:

- Data transparency
- Secure storage
- Accurate data management

Blockchain is a decentralized network where all users have access to the data available on it. For instance, any transaction/movement made by the organization can be seen by all users, making the process completely transparent. Any exchanges made between the organizations can be recorded and can be made available to users so that they can track their data movement. Its decentralized nature will make sure that systems are not dependent on one central authority. Instead, it is distributed across the nodes or members on the network for various approvals or acceptance, making it more trustworthy. The most interesting and essential feature of Blockchain is its immutability. Once a record is added to the network, the consent of a user cannot be modified. If there is a change of consent, it can be added one more time as a different block. Combining or mapping data from all sources is one of the most difficult tasks and can be done using Blockchain. Data can be aggregated in Blockchain using a distributed hash table (DHT) [22]. The encrypted consent data created by DHT can be stored on Blockchain, and the actual data can always be retrieved easily, which also resolves the size issue of the Blockchain, as more data cannot be stored in a block.

Another main application of Blockchain is identity management, where user’s data can be validated by an organization. For instance, an educational organization can validate a student’s final degree and store it on Blockchain, which can also be used to validate user data so that accurate data can be collected and stored. Therefore, using Blockchain, the consent management process can be made easier and more compliant.

## 4. Research Methodology

**Figure 2:**
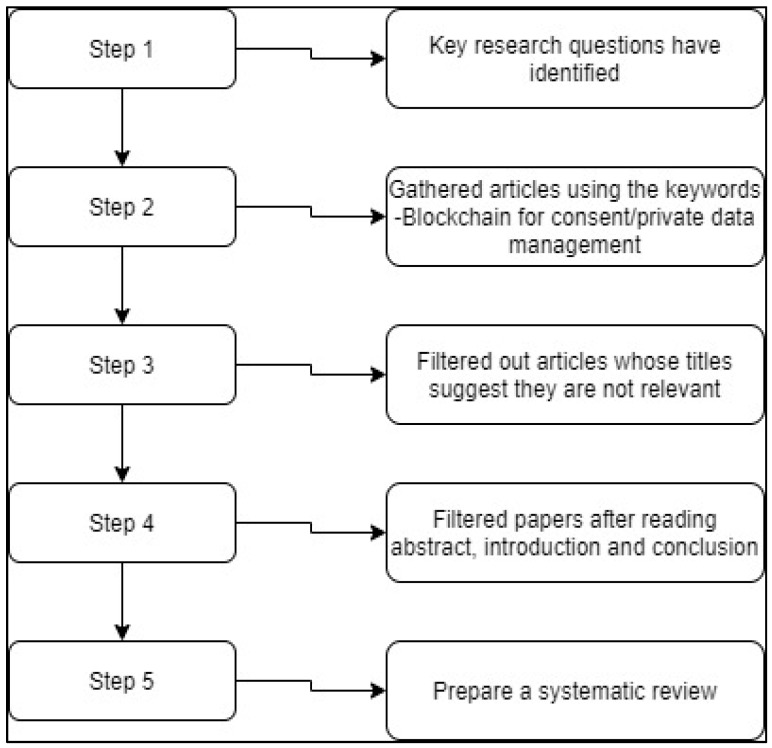
The process followed to conduct this review.

We also followed the recommendations for a systematic review of the literature [23] and the criteria outlined in the PRISMA statement [9]. In the first step, we have identified the critical research questions that would help us to understand what level blockchain-based technologies (related to consent/privacy data management) were built. The questions help us in examining the issues and constraints. The basic steps involved in conducting a systematic review are shown in the below Figure 2.

### Description of Research Objectives

We identified four research questions that we wanted in the review.

RQ1: How does Blockchain protect user privacy and data consent across different sectors?
  - This is one of the primary questions in this research to understand how Blockchain is used to implement consent management in various sectors such as healthcare, education, IoT, etc. We have reviewed multiple research papers and articles to understand different problems that blockchain applications could solve as PII (Personal Identifiable Information) policies went through a lot of change after the introduction of GDPR.
RQ2: What Blockchain-based applications have been built of the described use cases?
  - Although this technology was introduced in 2008 by the enigmatic Satoshi Nakamoto as an essential technology for Bitcoin, it is still a relatively new technology in other sectors such as healthcare, IoT, and education. Many application proposals were given in the scientific reviews, but very few discussed the working prototypes. Therefore, it is vital to learn and understand the real-world implementations.
RQ3: How are the limitations of current solutions addressed?
  - Here our objective is to find out the limitations of the technology based on the prototypes that have been developed? We will also examine the steps taken to avoid such limitations.
RQ4: What are the major challenges for future research?
  - With the introduction of GDPR rules in the EU, we have many restrictions on the use of PII. With this question, we identify the research gaps and challenges that need to be addressed so that the applications follow the new rules and policies.

**Figure 3:**
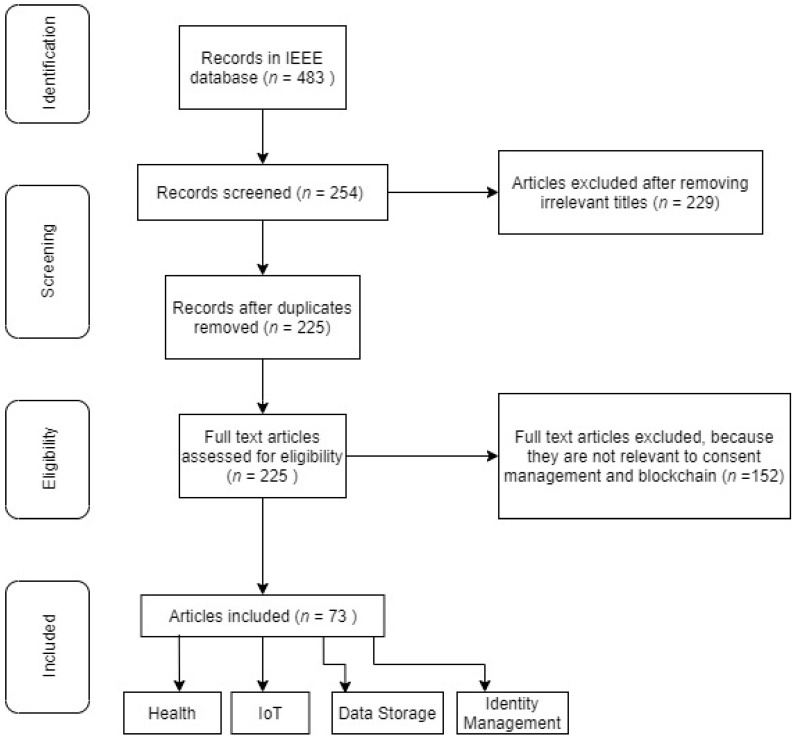
A PRISMA flow of this systematic review.

Figure 3 shows the PRISMA flow of this systematic review.

In the next step, we have searched for research papers in a reputable database (IEEE) with the keywords “Blockchain for consent management” and “Blockchain for private data management” and collected research papers. In the following step of the filtering, we have removed those documents whose title indicated that they were not of relevance to the topic. A few papers, for instance, were about Blockchain, but not related to the field of consent management.

In the next step, the following exclusion criteria were used after reading the abstract thoroughly:

- If the paper is not in the English Language
- Duplicate papers were removed
- If the paper is not related to consent/private management

In the next step, we have analyzed the remaining papers thoroughly and divided them into categories such as healthcare, IoT, etc. As shown in Table 1 below, we have also extracted the following data from the papers.

## 5. Results

The findings of the systematic review are summarized in this section. Using the mentioned keywords, we have gathered 483 research papers where we filtered out the paper whose titles suggested that they were not relevant to the research. After this step, we were left with 254 papers of various researchers, which were combined into a single file to check for duplicates; we found around 29 duplicate papers and removed them from the list. After reading the abstract, introduction, and conclusion extensively, we had to remove 152 papers, as they had nothing to do with Blockchain for consent/privacy management. The outcome was 73 papers selected. The full list of final papers is shown in Table 2 below.

### 5.1. Analysis of the Papers

**Figure 4:**
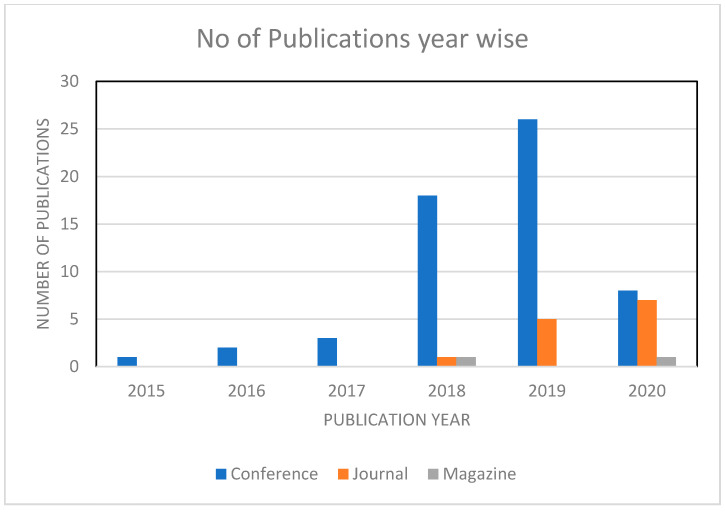
The number of publications per year.

We found that more relevant papers were published after 2015, and very few papers were published in the years 2016 and 2017. Additionally, 27% of the papers were published in 2018, and most papers, which amounts to 43%, were published in 2019. From 2020 onwards, we have 22 percent of the papers that are considered for review. The following Figure 4 gives us more details on the distribution of papers on a year-to-year basis.

**Figure 5:**
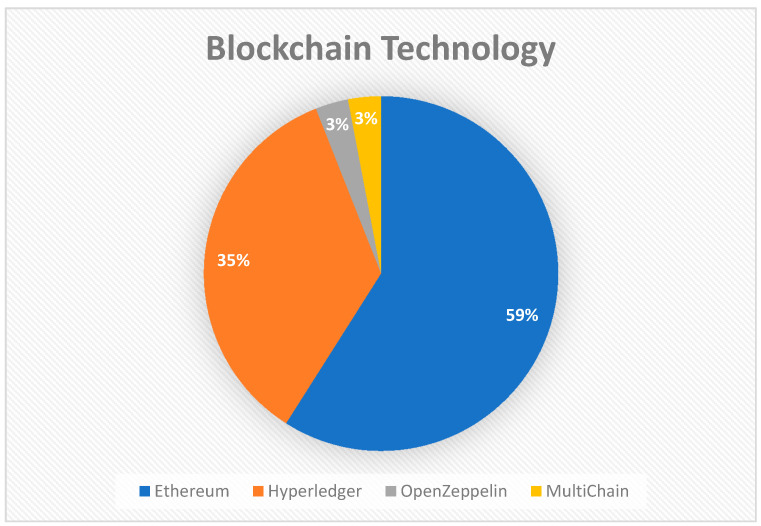
Choice of Blockchain platforms.

Almost a few percent of the papers only discussed the technical part of the technology without a working prototype, out of all the papers which discussed an implementation, the most used technology is Ethereum, followed by Hyperledger Fabric. Figure 5 below shows the percentage of the technologies used for implementation.

**Figure 6:**
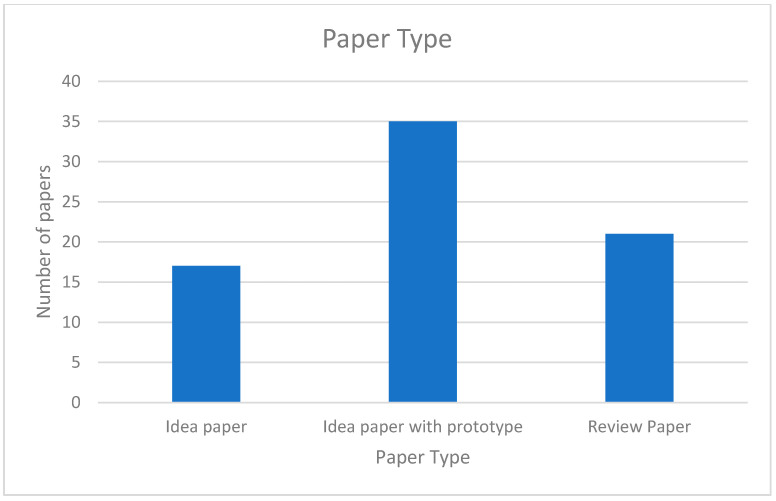
Paper types.

The papers we have reviewed included idea papers, idea papers with prototypes, and review papers. as shown in Figure 6. Many papers had technical details of the prototype implementation. Some papers included design ideas but no implementation or evaluation details. Review papers focused on various applications in the field.

**Figure 7:**
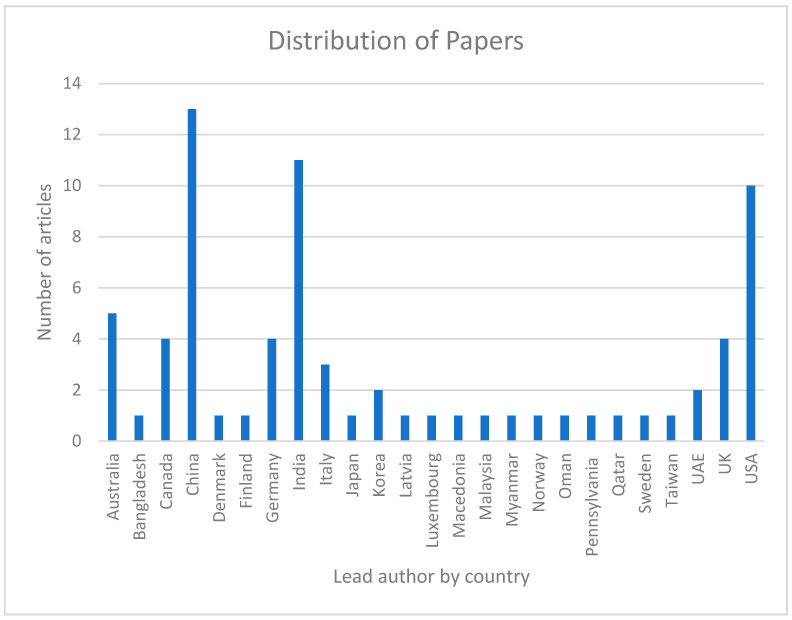
Distribution of papers by country of the lead author.

Finally, we have used the locations (countries) of those organizations of which writers of selected papers are associated to get an idea of the regional distribution of research group members interested in blockchain research. We have taken the lead author country details if the authors are from different countries. The geographical distribution of the papers chosen for the study is shown in Figure 7. From the figure, we can observe that authors in China, India, and the USA published most of the papers. They are followed by Australia, Canada, Germany, the UK, and Italy, respectively. Authors from the rest of the countries submitted less than two research papers.

### 5.2. Classification of the Papers

Most of the selected papers were conference papers, and the next significant type of selected papers are journal type papers. We have also identified the industry sectors such as healthcare, IoT, identity management and storage, to classify the use cases of the papers and have discussed a few main sectors where Blockchain is implemented.

#### 5.2.1. Healthcare

Researchers have identified that the healthcare industry is one of the primary industries that can benefit from Blockchain technology. As it follows the distributed ledger concept, medical records can be shared easily between the hospitals/doctors/researchers for various reasons, which can easily be used for managing a patient’s data. Counterfeiting medicines are a big concern for the pharmaceutical industry [49]. Health research funding studies have shown that 10% to 30% of drugs sold in developing countries are counterfeit [96]. The WHO reports that 16% of counterfeit products have the wrong ingredients, while 17% have an imprecise amount of necessary ingredients [49]. The use of these can lead to severe health problems and sometimes cause death. Blockchain can be used to avoid this, as it is digitally timestamped, and information cannot be changed/tampered with.

As stated, the main advantage of Blockchain in the healthcare industry is that it can be used to track and manage the patient’s data. Severe diseases require a personalized treatment, and a person cannot consult a single doctor for every disease. Hence, Blockchain is a platform where we can share this sensitive data, and we can make sure that it is safe and secure. Asaph Azaria and the team have proposed a framework called MedRec to share the medical information, which also has storage solutions [25]. They also introduced the incentives concept by asking the stakeholders to participate in the network and verify as miners [25]. S. Amofa also discussed the importance of sharing the data between doctors and built a framework using Ethereum [41]. In [37], Blockchain was implemented in a developing country, Nepal, where, with the implementation, there was a financial gain of 63%.

While it is important to share the data between the organizations, it is also important for researchers to access and analyze health information. Vero Estrada-Galinanes and K. Wac discussed such importance in [48] and proposed the development of an Open Health Archive (OHA), a shared forum for maintaining and archiving personal health records. The importance of the research in the health care sector is discussed in the articles [75,88] by the respective authors. The research articles [77,80] discussed the importance of sharing the data in the Cloud, as PII is not allowed directly in Blockchain because of GDPR, and they have also explained it with their respective working prototypes.

The research papers [70,95] examined the importance of user control over the data, which is shared, and working models have been built using Ethereum and Hyperledger Fabric, respectively. Emergency access to health records is discussed in [50]; it can be achieved with help of smart contracts. The author suggested having a smart contract that will give access to information in case of an emergency for a limited period. SHEMB is an Ethereum-based solution that requires a third party in storing data using a symmetrically searchable encryption technique to speed up access to information using the patient’s search query [66].

#### 5.2.2. IoT

This is another field where Blockchain implementation can revolutionize the world. IoT applications are those that are connected to the Internet and can communicate with each other. Though there are many advantages of IoT devices, the main concern is that they collect sensitive information like our location, etc., and they can be used to make or understand the behavioral patterns of a user. Therefore, Blockchain technology integration with IoT devices will result in privacy issues being nullified.

The papers [26,45] discussed the implementation of Blockchain in the IoT field and the limitations that come along with it. Cha, S.C. and others have suggested the design of a blockchain linked gateway that preserves user privacy preferences for IoT devices on the blockchain network adaptively and securely [45].To make sure the IoT data is secure, Fariza Sabrina came up with a solution that discusses the privacy data challenge. An approach is a service-oriented approach and a mix of public Blockchain (with smart contracts) and local off-chain data for entitlement management and control [56]. The architecture is implemented using Ethereum and helps to secure confidential data. In the article authored by [53], the paper discusses the importance of trust while integrating Blockchain and IoT. Here, the implementation is done using Ethereum Framework, and the decentralized network is laid between the patient, insurance provider, and hospital instead of a traditional centralized network. The main disadvantage of a centralized network is that it is a time taking process, and there is no proper transparency.

#### 5.2.3. Identity Management

Usually, personal identity is verified using documents such as Social Number, license, and passport. However, there is hardly an efficient equivalent method for protecting online identities [49]. Using Blockchain, a digital identity/ID can be created and can be used for online transactions instead of using real identities. This way, we can eliminate the possibility of online fraud. Blockchain-based identity management solutions may allow customers to access and validate online purchases by simply using an authentication app instead of using a username and password or biometric system [97]. Additionally, the self-sovereign identity concept in Blockchain is introduced by D. Baars to eliminate identity theft [98]. A new way of storing important documents related to education is explained by Alan Colman and the team in [34], and it is implemented using Ethereum. The author proposed a framework that can store the data and authenticate the education-related documents where the authentication of documents is done by the University or College and stored on the Blockchain. As the data in Blockchain cannot be tampered with, we can always request authentication. Paul Dunphy et al. introduced a framework for identity management, using distributed ledger technology to improve decentralization, accountability, and user control [36].

#### 5.2.4. Storage

Though direct PII cannot be stored in a Blockchain network, this can be used to store encrypted data. One of the main applications of Blockchain is the ability to combine with third parties like Cloud, etc., to store the data. This enables users to have data transparency and the ability to track the usage of their data. In [68], they introduced a new technique called interest groups, where each group will hold to a field data. Now the groups can sell/borrow/rent the data that they hold. They also spoke about the rewards that could be given for a group that offers the most relevant data. Data transparency is the main feature. As stated before, the size of a block and GDPR make it difficult to store data on Blockchain. Therefore, Saqib Ali and others came up with the idea of Pinger [42]. It stores the metadata of the files, while the original files are stored in various locations with the aid of a DHT (distributed hash table). Using Ethereum and IPFS (InterPlanetary File System), M. Alessi and the team came up with a prototype [34], which can store personal data and also has requested data service.

These are the main classifications that we found in the process. There are other fields like agriculture, where Blockchain is used. The importance of farmer’s consent using Blockchain is mentioned in [99]. Consentio [100] is a general consent management system that is built on Hyperledger Fabric that can be used for various cases such as EHR (Health), small infrastructure (IoT), and social media. Few papers also discussed the main applications, challenges, and issues [27,60,63,64,81].

## 6. Discussion

In this section, we utilize our findings to cross-examiner the research questions we have identified in Section 4. We provide a discussion of our analysis and address the limitations of the review.

RQ1: How does Blockchain protect user privacy and data consent across various sectors?

Blockchain is used in various types of industries—healthcare, IoT, Identity Management, etc. The most common use cases are mentioned below in Table 3.

RQ2: What Blockchain-based applications have been built of the described use cases?

Although the concept of Blockchain was introduced in 2008, it is still a new technology for other sectors except for Finance. Many of the papers presented technical implementation; very few papers had a practical implementation, and few of those details are given in Table 4.

Out of the implementations that have been developed, we have observed that Ethereum is the most used framework, and it is followed by Hyperledger Fabric. The concept of Smart Contracts in Ethereum is one of the main reasons it is the most used Framework.

RQ3: How are the limitations of current solutions addressed?

The main challenges are interoperability, block size, regulations, response time, and the GDPR rule “right to be forgotten”. Interoperability is a big challenge that is being faced, as we cannot operate between two frameworks, and the integration of Blockchain with legacy systems is still an issue. After interoperability, the size of the network is a concern, as the Bitcoin network is around 270 GB, and Ethereum size already crossed 1 TB [101]. The main challenge with the size of these networks is the transaction speed and validation process. Due to the increasing size of the networks, the main challenge is to the validators/contributors who need to spend more money to make their nodes run. Not only the cost but also the response time of the network is impacted due to this. Now bigger blocks are very much challenging to running the network, whereas smaller blocks are feasible to run and work well with a third-party solution like Cloud. As a decentralized system, it does not have standard regulations making it vulnerable. Coming to another challenge in Blockchain technology is the GDPR rules that were introduced in 2018 to protect the PII of European Nations. It means that a person is entitled to ask the data to be erased. But Blockchain being immutable, it is impossible to erase data from the network. Finally, there are a few security concerns with the existing applications. A potential breach could leak all the user’s data, as there have been several attacks on certain Blockchain applications. There is also a risk of identifying a user when there is adequate information available on the network.

There are few networks such as Cosmos [102] and Polkadot [103] that came up with the solution for interoperability. As a part of the Cosmos network, independent networks (called zones) are connected through the Cosmos Hub. Additionally, Ethereum 2 (also known as Serenity) solves a few critical issues with the introduction of concepts such as subchain and POS (Proof of Stake) [104]. These features now enable a person to maintain small transactions without mining, which reduces the bloating problem. With the help of the subchain, now the transactions will be cheaper and quicker. Additionally, the 51% percent attack can be avoided with the POS concept, as a person/miner with more than 51 percent of control would not want to attack their network. Additionally, erasing data from local storage is possible, as we do not store the PII on the network. This has been explained by M. Florian in [63]. The author suggested an approach where we can completely delete the undesirable data from local nodes. By this, we can ensure the GDPR rule, which is the right to be forgotten. Additionally, only a few nodes can participate in the consensus and validation processes to boost the efficiency of the system and increase the processing speed [105]. Currently, more extensive research is going to improve the current standards and conditions of Blockchain technology to make it more useful. Permissioned Blockchain should be used instead of the public Blockchains to avoid attacks and sharing of the data. To protect the user information on the network, encryption technologies are being used, and techniques are also to be updated periodically.

RQ4: What are the major challenges for future research?

Blockchain solves many real-world problems, such as data authentication, data storage, and data privacy. It is still in the early stages of implementation, as there are not many working prototypes at this moment. Therefore, extensive research is going in every field to implement this technology, especially in the field of data management, as we believe that it can eliminate the process of filling up forms, as the data can be shared between any departments/organizations securely. More working models are needed to come up with the best practice to implement for managing the data; for this reason, the prototypes must be tested thoroughly. We believe it will play a crucial role in the future healthcare, IoT, data storage, identity management, and Telecom sectors, respectively. Additionally, to make sure the new working prototypes are approachable to the people, there is a need for a few regulations to be introduced.

One of the main focuses is also on the interoperability of the frameworks. Apart from interoperability, the other main challenges that need to be worked upon are security and privacy; the speed of adding blocks to a network is one constant area that also needs to be checked. These are a few areas where there are open research questions. It is also highly essential that Blockchain complies with all the regulations set by GDPR. By meeting all the requirements, it will give a boost to the companies to integrate into their existing solutions.

### Limitations of the Review

Despite the various benefits of conducting a systematic analysis, certain drawbacks need to be considered: bias in the collection, bias in publication, imprecision in the extraction of data, and misclassification [23]. To eliminate the bias, we investigated the papers irrespective of novelty or unremarkable outcomes. To achieve a maximum possible number of papers, an efficient search protocol was developed for research papers, followed by a search with all alternative keywords. Moreover, criteria for inclusion and exclusion have been established to ensure that the papers included are focused on research topics that are well linked to the research goals.

Inaccuracies in the extraction of data and misjudgments may be attributed to the reviewer’s failure to accurately retrieve information and data from its records. We have used an Excel sheet throughout the process to make sure all the extracted data have been properly recorded.

## 7. Conclusions and Future Work

Blockchain is an evolving technology that will revolutionize the information technology world, because it can be applied in many applications domains where an immutable ledger is useful. To evaluate the applications of blockchain to consent management and private data management, we have conducted this systematic review using the PRISMA guidelines to identify the current solutions, limitations and research challenges. Overall, 73 papers were studied in detail to perform this review.

Our study indicates that Blockchain has been implemented in various fields such as healthcare, IoT, data storage, and management. Many prototypes have been developed and are already tested in real-world scenarios. More research is being conducted on this, and in the future, more research will be done to understand and evaluate the implementations. Further research is aimed at solving the issues associated with the use of blockchain technology such as scalability, latency, interoperability, privacy, and security.

## Figures and Tables

**Table 1:** Classification of papers.

Data Item	Description
Authors	Authors of the paper
Title	Title of the paper
Year	Paper year publication
Publication Type	Journal/Conference/Magazine
Prototype	Yes or No

**Table 2:** Selected papers.

Author	Year	Publication Type
Guy Zyskind. et al. [24]	2015	Conference
Asaph Azaria. et al. [25]	2016	Conference
Marco Conoscenti. et al. [26]	2016	Conference
Elena Karafiloski. et al. [27]	2017	Conference
Fangfang Dai. et al. [28]	2017	Conference
Shinsaku Kiyomoto. et al. [29]	2017	Conference
Jatinder Singh. et al. [30]	2018	Conference
Kaiwen Zhang. et al. [31]	2018	Conference
Klaus Zaerens [32]	2018	Conference
Liping Liu. et al. [33]	2018	Conference
M. Alessi. et al. [22]	2018	Conference
Mohammad Jabed Morshed Chowdhury. et al. [34]	2018	Conference
Nasr Al-Zaben. et al. [35]	2018	Conference
Paul Dunphy. et al. [36]	2018	Magazine
Pranita Upadhyaya. et al. [37]	2018	Conference
Qianyi Dai. et al. [38]	2018	Conference
R. ANGELINE [39]	2018	Conference
Reza Soltani. et al. [40]	2018	Conference
Sandro Amofa. et al. [41]	2018	Conference
Saqib Ali. et al. [42]	2018	Conference
Sebastian Friebe. et al. [43]	2018	Conference
Shanto Roy. et al. [44]	2018	Conference
Shi-Cho Cha. et al. [45]	2018	Journal
Shirley Crompton. et al. [46]	2018	Conference
Sushmita Ruj. et al. [47]	2018	Conference
Vero Estrada-Galinanes. et al. [48]	2018	Conference
Ahmed Afif Monrat. et al. [49]	2019	Journal
Ahmed Raza Rajput. et al. [50]	2019	Journal
Ajay Kumar Shrestha. et al. [51]	2019	Conference
Anang Hudaya Muhamad Amin. et al. [52]	2019	Conference
Bhabendu K. Mohanta. et al. [53]	2019	Conference
Bhabendu Kumar Mohanta. et al. [54]	2019	Conference
Dipti Ashok Belurgikar. et al. [55]	2019	Conference
Fariza Sabrina [56]	2019	Conference
Florian Zemler. et al. [57]	2019	Conference
Hongzhi Li. et al. [58]	2019	Journal
Huang Bowen. et al. [59]	2019	Conference
Hye-Young Paik. et al. [60]	2019	Journal
Ingo Weber. et al. [61]	2019	Conference
Malak Parmar. et al. [62]	2019	Conference
Martin Florian. et al. [63]	2019	Conference
Nilima D. Pai. et al. [64]	2019	Conference
Nitin Sukhija. et al. [65]	2019	Conference
Nitish Andola. et al. [66]	2019	Conference
Ping Zhong. et al. [67]	2019	Conference
Ronald Doku. et al. [68]	2019	Conference
Rujuta Shah. et al. [69]	2019	Conference
Shadan Ghaffaripour. et al. [70]	2019	Conference
Taylor Hardin. et al. [71]	2019	Conference
Thang X. Vu. et al. [72]	2019	Conference
Thorsten Weber. et al. [73]	2019	Conference
Tiffany Hyun-Jin Kim. et al. [74]	2019	Conference
Vinay Mahore. et al. [75]	2019	Conference
Wie Liang Sim. et al. [76]	2019	Conference
Xiaoguang Liu [77]	2019	Journal
Yiheng Liang. et al. [78]	2019	Conference
Yongseon Ji. et al. [79]	2019	Conference
Abdulbadi Sabir. et al. [80]	2020	Conference
Emanuele Bellini. et al. [81]	2020	Journal
James P. Howard. et al. [82]	2020	Magazine
Jãnis Grabis. et al. [83]	2020	Conference
Javed Ahmed. et al. [84]	2020	Conference
Kai Fan. et al. [85]	2020	Journal
Ma Zhaofeng. et al. [86]	2020	Journal
Manaf Zghaibeh. et al. [87]	2020	Journal
Mingxiao Du. et al. [88]	2020	Journal
Nguyen Binh Truong. et al. [89]	2020	Journal
Randhir Kumar. et al. [90]	2020	Conference
Richa Gupta. et al. [91]	2020	Conference
Saifull ah Khan. et al. [92]	2020	Conference
Shahriar Badsha. et al. [93]	2020	Conference
Soe Myint Myat. et al. [94]	2020	Conference
Yan Zhuang. et al. [95]	2020	Journal

**Table 3:** Use cases in various sectors.

Field	Usage
Healthcare	To share the medical information securely between doctors/organizations/researchers
IoT	To have the user’s privacy preference for IoT Devices
Identity Management	Management of identity verification
Storage	Tamper-proof activity monitoring and data access control protection

**Table 4:** Existing applications.

Field	Application
Healthcare	MedRec [25], SHEMB [66]
IoT	Blockchain-enabled Security Architecture [56]
Identity Management	Blockchain as a Notarization Service [34]
Storage	Pinger [42], Pledge [68]

## Data Availability

Not applicable.
